# A Randomized Controlled Trial of Sertraline in Young Children With Autism Spectrum Disorder

**DOI:** 10.3389/fpsyt.2019.00810

**Published:** 2019-11-06

**Authors:** Laura A. Potter, Danielle A. Scholze, Hazel Maridith B. Biag, Andrea Schneider, Yanjun Chen, Danh V. Nguyen, Akash Rajaratnam, Susan M. Rivera, Patrick S. Dwyer, Flora Tassone, Reem R. Al Olaby, Nimrah S. Choudhary, Maria J. Salcedo-Arellano, Randi J. Hagerman

**Affiliations:** ^1^Medical Investigation of Neurodevelopmental Disorders (MIND) Institute, UC Davis Health, Sacramento, CA, United States; ^2^Department of Pediatrics, University of Wisconsin School of Medicine and Public Health, Madison, WI, United States; ^3^Department of Pediatrics, UC Davis Health, Sacramento, CA, United States; ^4^College of Psychology, California Northstate University, Elk Grove, CA, United States; ^5^Institute for Clinical and Translational Science, University of California, Irvine, Irvine, CA, United States; ^6^Department of Medicine, University of California, Irvine School of Medicine, Orange, CA, United States; ^7^Case Western Reserve University School of Medicine, Cleveland, OH, United States; ^8^Department of Psychiatry and Behavioral Sciences, UC Davis Health, Sacramento, CA, United States; ^9^Department of Psychology, University of California, Davis, Davis, CA, United States; ^10^Department of Biochemistry and Molecular Medicine, UC Davis Health, Sacramento, CA, United States; ^11^College of Health Sciences, California Northstate University, Rancho Cordova, CA, United States

**Keywords:** sertraline, autism spectrum disorder, controlled trial, targeted treatment, nonsyndromic autism spectrum disorder

## Abstract

**Objective:** Selective serotonin reuptake inhibitors like sertraline have been shown in observational studies and anecdotal reports to improve language development in young children with fragile X syndrome (FXS). A previous controlled trial of sertraline in young children with FXS found significant improvement in expressive language development as measured by the Mullen Scales of Early Learning (MSEL) among those with comorbid autism spectrum disorder (ASD) in *post hoc* analysis, prompting the authors to probe whether sertraline is also indicated in nonsyndromic ASD.

**Methods:** The authors evaluated the efficacy of 6 months of treatment with low-dose sertraline in a randomized, double-blind, placebo-controlled trial in 58 children with ASD aged 24 to 72 months.

**Results:** 179 subjects were screened for eligibility, and 58 were randomized to sertraline (32) or placebo (26). Eight subjects from the sertraline arm and five from the placebo arm discontinued. Intent-to-treat analysis showed no significant difference from placebo on the primary outcomes (MSEL expressive language raw score and age equivalent combined score) or secondary outcomes. Sertraline was well tolerated, with no difference in side effects between sertraline and placebo groups. No serious adverse events possibly related to study treatment occurred.

**Conclusion:** This randomized controlled trial of sertraline treatment showed no benefit with respect to primary or secondary outcome measures. For the 6-month period, treatment in young children with ASD appears safe, although the long-term side effects of low-dose sertraline in early childhood are unknown.

**Clinical Trial Registration:** www.ClinicalTrials.gov, identifier NCT02385799.

## Introduction

Autism spectrum disorder (ASD) is a behaviorally defined developmental disorder characterized by deficits in social communication and interaction in conjunction with the presence of restricted, repetitive patterns of behavior (1). Speech delay and other language deficits are common in early development. ASD is understood to have many causes, including an estimated over 1,000 gene mutations that can lead to deficits in synaptic plasticity, neuronal migration, transcription and translation changes, and many other processes important for synapse development and central nervous system (CNS) connectivity (2). Currently, the Centers for Disease Control and Prevention reports that 1 in 59 children at age 8 years in the general U.S. population has ASD (3). Known specific causes accounting for more than 2 to 3% of all ASD cases include fragile X syndrome (FXS) and tuberous sclerosis, although many more single gene mutations, duplications, or deletions can cause 1% or less of cases. In individuals with ASD compared to neurotypical controls, higher rates of putative functional *de novo* mutations are observed (1), and whole genome sequencing can identify genetic mutations in up to 30% of those with ASD (4). As many as 50% of *de novo* mutations resulting in ASD appear to occur in genes regulated by or associated with FMRP, the protein lacking in patients with FXS due to methylation of the *FMR1* gene on the X chromosome, suggesting a potential overlap in beneficial treatments between FXS and ASD (5).

There is growing evidence of a sensitive period in the development of young children with neurodevelopmental problems, during which the brain is rapidly developing and thus may be more susceptible to intervention with targeted treatments (6–12). Targeted behavioral interventions in ASD, such as the Early Start Denver Model, demonstrate improvements in behavioral and developmental symptoms as well as electroencephalogram parameters for children under 5 years, compared with community intervention (13, 14). Pharmacological interventions in children under 6 years with ASD may also be warranted, but few clinical trials have been conducted to date.

Disruption of serotonergic development has long been suggested as a causal factor in the altered brain function and behaviors seen in neurological disorders including autism. Positron emission tomography scanning has demonstrated that young children with ASD show lower levels of serotonin production compared with neurotypically developing controls during the first 5 years of development, when rapid synapse formation typically takes place (15). Further, using lymphoblastoid lines, a metabolomics study of several forms of ASD revealed a deficit of the enzymes needed to convert tryptophan into serotonin (16). Such evidence has spurred research into whether selective serotonin reuptake inhibitors (SSRIs) like sertraline could reverse the neurophysiological changes observed in ASD.

Sertraline is currently approved by the Food and Drug Administration for the treatment of Obsessive Compulsive Disorder in children aged 6 to 17 years, as well as several psychological disorders including major depressive disorder and social anxiety disorder in adults. In clinical practice, sertraline has been used to treat self-injury, aggression, anxiety, and depressed mood in individuals with ASD. Sertraline has been proposed as an optimal SSRI for ASD because of its lesser activating effects compared to other psychotropic medications and its minimal interaction with the metabolism of other medications compared with other SSRIs.

Animal studies have further investigated the effects of SSRIs like sertraline on various biomarkers related to early development in ASD and other neurological disorders. There is evidence in mouse models of Down syndrome that SSRIs stimulate brain-derived neurotrophic factor (BDNF) when given early in development (17). BDNF levels are correlated with late-phase long-term potentiation in mice, synapse regulation and cognitive function in adult humans, and behavioral changes such as susceptibility to anxiety and aggression in both mice and humans (18, 19). Clinical evidence suggests altered serum concentrations of BDNF in children with ASD compared to controls, though CNS levels are not known (20). Pro-cognitive and neuroprotective effects were seen in wild-type mice treated with sertraline, with a supposed mechanism in which sertraline’s upregulation of BDNF in turn upregulated serotonin, dopamine, and gamma-aminobutyric acid (GABAergic) systems (21). In the knockout FXS mouse model, BDNF treatment also rescued synaptic plasticity (22). Abdallah et al. showed that matrix metalloproteinase-9 (MMP9), an endopeptidase implicated in early postnatal synaptogenesis (7, 23), was elevated in the amniotic fluid of ASD cases compared to controls. Additionally, increased levels of brain serotonin in mice have been shown to correlate with MMP9 activity, making both BDNF and MMP9 possible biomarkers of treatment with SSRIs in ASD (24, 25).

Few clinical trials of SSRIs and other serotonin-influencing drugs have been conducted to date in children under 6 years with idiopathic or syndromic ASD, perhaps due to the overall negative results of studies of SSRIs in their older child and adolescent counterparts to date (26); however, those trials in young children that have been published have shown promising results (27–29). In 2012, a retrospective study of low-dose sertraline in 45 children with FXS with and without ASD aged 12 to 50 months demonstrated significant improvements in the trajectory of receptive and expressive language in those on sertraline compared with those not treated with sertraline (27). In prespecified secondary analyses, Chugani et al. showed that low-dose buspirone, a partial serotonin receptor agonist, in children aged 2 to 6 years with ASD could be an effective treatment to reduce restrictive and repetitive behaviors in conjunction with behavioral interventions (28). Greiss Hess et al. demonstrated the benefit of low-dose sertraline (2.5 to 5.0 mg/day) in children with FXS with and without ASD aged 2 to 6 years with improvements in secondary outcomes of visual reception, fine motor, and cognitive T score sum on the Mullen Scales of Early Learning (MSEL) (29). The children receiving sertraline also performed better than those in the placebo group on a passive-viewing eye tracking (PVET) task of receptive vocabulary post-treatment compared to baseline (30). Moreover, polymorphisms of several genes involved in the serotonergic pathway, such as BDNF and 5-HTTLPR (serotonin-transporter-linked polymorphic region), were identified as potential predictors of response to sertraline treatment for these children (31). In *post hoc* analysis, those with both FXS and ASD demonstrated significant improvements on sertraline compared to placebo in expressive language development on the MSEL (29). These results prompted the authors to carry out the study reported here to investigate whether sertraline has a similar benefit in young children with nonsyndromic ASD.

## Materials and Methods

### Participants and Design

This was an exploratory first trial of sertraline in children with non-FXS ASD aged 2 to 6 years using a randomized, double-blind, placebo-controlled, parallel two-arm design between April 2015 and July 2018. Inclusion criteria included documentation of ASD as verified using both *Diagnostic and Statistical Manual, 5th edition (DSM-5)* and Autism Diagnostic Observation Schedule, 2nd edition (ADOS-2) criteria, age between 24 and 72 months, stable medications (including antiepileptics, antipsychotics, and clonidine) in the 2 months prior to enrollment, and concurrent enrollment in at least one community or school intervention for ASD. Changes in concomitant medications and interventions were discouraged unless medically necessary during the trial. Exclusion criteria included current or past SSRI treatment, diagnosis of the FXS full mutation, or any other serious co-morbid medical disorders affecting brain function and behavior, including uncontrolled seizures. Molecular testing to rule out FXS diagnosis was carried out by PCR approach as described in Tassone et al. (32).

The UC Davis Investigational Drug Services independently carried out randomization to sertraline or placebo. The placebo was formulated as a clear, colorless solution and contained menthol so as to match both the appearance and smell of the liquid sertraline. The study drug was administered orally in liquid form (20 mg/ml), and dose was assigned based on age at enrollment: subjects under 4 years received sertraline or placebo liquid in a dose of 2.5 mg/day (0.125 ml) for the duration of the trial, and subjects 4 years or older received 5.0 mg/day (0.25 ml). These doses were based on those used in previous studies in young children with FXS (27, 29).

### Assessments

With the exception of the Clinical Global Impression Scale—Severity (CGI-S; completed only at baseline) and the Clinical Global Impression Scale—Improvement (CGI-I; completed only at 3 months and EOT), all assessments described forthwith were conducted in clinic at baseline and end-of-treatment (EOT) visits.

Subject assessments included the four subtests of the MSEL, which were administered to assess changes in visual reception, fine motor, receptive language, and expressive language abilities. The Preschool Language Scales—Fifth Edition (PLS-5) was used to further evaluate auditory comprehension and expressive communication skills. A 30-trial passive-viewing eye tracking (PVET) task was also administered as described in Yoo et al. (30). In PVET trials, subjects looked at images of a target word and a distractor; an audio recording (e.g., “Look at the *ball*”) was used to indicate the target.

Caregiver assessments included the Visual Analog Scale (VAS), measuring parent-reported severity of three target behaviors: obsessive-compulsive behavior/anxiety, language/communication, and hyperactivity/hyperarousal/aggression. Other caregiver assessments included the Vineland Adaptive Behavior Scales—Second Edition (VABS-II) survey interview to assess level of adaptive functioning; the Aberrant Behavior Checklist—Community (ABC-C) to measure the severity of certain problematic behaviors; the Preschool Anxiety Scale—Revised (PAS-R) to assess symptoms of anxiety and fears; the Sensory Processing Measure—Preschool (SPM-P) to measure sensory processing difficulties; and the Social Responsiveness Scale (SRS-2) to measure ASD symptom severity in natural social settings.

A 3-month supply of study drug and a caregiver dosing diary were dispensed at baseline and again at 3 months, and both remaining drug and diary were collected at subsequent visits to measure compliance. Safety monitoring consisted of a physical examination at every visit, weekly telephone calls in the first month and monthly calls thereafter, and baseline and EOT standard blood laboratory tests, including comprehensive metabolic panel and complete blood count with differential. When blood volume allowed, BDNF and MMP9 levels were measured at both baseline and EOT.

### Statistical Analyses

The study design included two primary outcomes: MSEL expressive language (EL) raw score and MSEL age equivalent combined (AEC) score (an average of visual reception, fine motor, receptive language, and EL subtest age equivalent scores). The primary outcome measures were determined based on the prior study of sertraline in young children with FXS (29). Prespecified efficacy analyses were based on the analysis of covariance (ANCOVA), adjusted for corresponding baseline score. All other measures and associated analyses were secondary/exploratory. Data from the PVET task were analyzed using the eyetrackingR package (33). The proportions of fixation duration to the target and distractor word areas of interest were calculated during a window spanning 2,300–3,800 ms (i.e., 300–1,800 ms following audio onset of the target word). PVET trials were excluded from the analysis if participants did not fixate to either the target or distractor area of interest for at least 25% of the time during the analysis window; recordings were also excluded if usable data were available on fewer than 25% of trials. T-test was used for CGI-I. All other secondary analyses were based on the ANCOVA model for continuous outcomes with baseline measures and were implemented in SAS software version 9.4. Adverse events (AEs) were summarized by severity, relation to drug, and resolution status (ongoing *vs*. not ongoing). Student’s t-test and Fisher’s exact test were applied to continuous and categorical demographic variables, respectively. Tests for primary efficacy analyses were at adjusted significance level 0.025, while all other tests (exploratory) were at level 0.05. *Post hoc* analysis exploring whether baseline BDNF or MMP9 level or change in BDNF were associated with differential outcome improvement were based on regression model with the addition of each of these variables and the interaction with treatment term. The ANCOVA assumes a continuous (interval level) outcome, and it should be noted that this assumption does not in fact hold for the raw scores in these analyses. Also, distributions of variables were examined graphically, and where appropriate sensitivity analyses based on log-transformation were conducted and results remained the same.

The study was designed with 80% power to detect a standardized effect size of approximately 0.68 in a 2-arm parallel design with the endpoint at 6 months with significance level of 0.025 for two primary measures. The required total sample size was 60 (30 per group), but resource limitations limited the number of randomized, analyzable patients to 47. Calculation of “observed” power has been shown to be scientifically invalid and was therefore not performed (34). Under the current design conditions, a reduced sample size of ∼47 (i.e., at study completion) affords 68% power (a reduction from the planned 80% power). We note that given that the study results suggest a potential effect size of 0.12, a future study would require 1,084 patients to detect such a small effect size. Because this small effect size is clinically not meaningful, such a future study would be ethically unacceptable.

## Results

### Subject Characteristics

**Figure 1:**
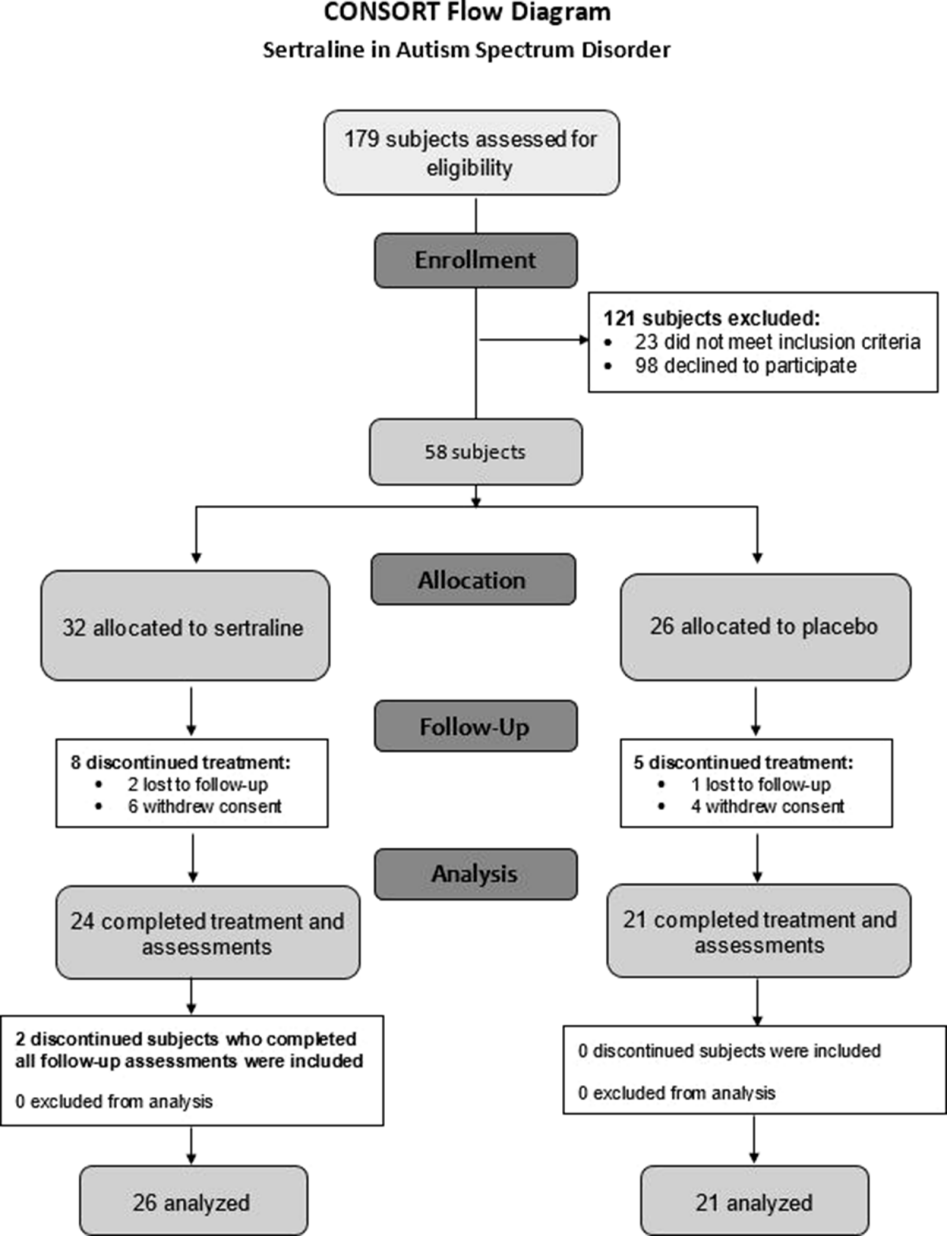
CONSORT flow diagram. CONSORT, Consolidated Standards of Reporting Trials.

Of the 179 subjects contacted regarding the study, 81 were assessed for eligibility, and 58 participants met inclusion criteria and agreed to participate (**Figure 1**). The 58 participants were randomized to the sertraline arm (n = 32) or placebo arm (n = 26). Baseline characteristics between groups are shown in **Table 1**. The majority of participants in the treatment arms (sertraline *vs*. placebo) were boys (78 *vs*. 81%), spoke English as their primary language (94 *vs*. 88%), and identified as Caucasian (59 *vs*. 69%). There were more non-Hispanic participants in the sertraline arm than the placebo arm (91 *vs*. 69%). The average (SD) age was 4.3 (0.8) and 3.7 (1.1) years in the sertraline and placebo groups, respectively. Level of education of the primary caregiver, ADOS-2 classification, and use of concomitant medication showed no significant difference between treatment groups. None of the subjects had a molecular diagnosis of FXS. A total of 13 subjects discontinued (8 from the sertraline arm and 5 from the placebo arm); of these, only 2 in the sertraline arm voluntarily returned to complete EOT assessments. Thus, 45 participants completed 6 months of treatment, and a total of 47 were analyzed.

**Table 1:** Baseline characteristics of study subjects.

Variable	Category	Sertraline (N = 32)		Placebo (N = 26)		P-value*
		N	%	N	%	
Age	Mean/SD	4.31	0.90	3.70	1.10	0.0475
MSEL DQ	Mean/SD	48.72	21.46	50.63	24.05	0.7790
MSEL DQ V	Mean/SD	35.84	21.89	42.58	31.24	0.4020
MSEL DQ NV	Mean/SD	53.01	21.95	53.31	22.25	0.9630
Sex	Male	25	78.13	21	80.77	1
	Female	7	21.88	5	19.23	
Primary language	English	30	93.75	23	88.46	0.6482
	Other	2	6.25	3	11.54	
ADOS-2	ASD	2	6.25	1	3.85	1
	Autism	30	93.75	25	96.15	
Race	Caucasian	19	59.38	18	69.23	0.6396
	Black	5	15.63	2	7.69	
	Asian	7	21.88	4	15.38	
	Other/unknown	1	3.13	2	7.69	
Ethnicity	Non-Hispanic/Latino	29	90.63	18	69.23	0.0496
	Hispanic/Latino	3	9.38	8	30.77	
Education	Unknown	8	25	9	34.62	0.5897
	Partial college	10	31.25	5	19.23	
	College degree	7	21.88	8	30.77	
	Graduate degree/	7	21.88	4	15.38	
	professional training					
Concomitant	No	29	90.63	24	92.31	1
Medication	Yes	3	9.38	2	7.69	

*Fisher’s exact test.

SD, standard deviation; MSEL, Mullen Scales of Early Learning; DQ, developmental quotient; V, verbal; NV, nonverbal; ADOS-2, Autism Diagnostic Observation Schedule, 2nd edition; ASD, autism spectrum disorder.

### Prespecified Primary Outcome Analysis

Prespecified intent-to-treat (as randomized) analyses were conducted for 2 designated primary outcome measures: MSEL EL raw score and MSEL age equivalent combined (AEC) score. Observed changes in scores at follow-up, adjusted for baseline scores, were not significantly different between sertraline and placebo groups (**Table 2**): EL raw score mean 20.3 (11.7) *vs*. 21.8 (14.3), F_1,44_ = 0.37, p = 0.547; AEC score mean 28.2 (14.2) *vs*. 30.3 (15.6), F_1,44_ = 1.11, p = 0.298.

**Table 2:** Primary efficacy results.

Variables	Sertraline						Placebo						
	Baseline			End-of-treatment			Baseline			End-of-treatment			
	N	Mean	SD	N	Mean	SD	N	Mean	SD	N	Mean	SD	P-value, F
MSEL EL raw score	32	18.19	10.39	26	20.31	11.70	26	19.69	11.69	21	21.81	14.29	0.5467, F_1,44_ = 0.37
MSEL AEC score	32	24.98	12.79	26	28.16	14.15	26	25.61	11.73	21	30.30	15.58	0.2976, F_1,44_ = 1.11

SD, standard deviation; MSEL, Mullen Scales of Early Learning; EL, expressive language; AEC, age equivalent combined.

### Secondary/Exploratory Outcome Measures and *Post Hoc* Analyses

Secondary measures included MSEL subtest raw and age equivalent scores in visual reception, fine motor, and receptive language; CGI-I at EOT; PLS-5 auditory comprehension and expressive communication raw and age equivalent scores; PVET; Visual Analog Scale for each of the three prespecified target behaviors at EOT; VABS-II; ABC-C; PAS; SRS; and SPM-P. Overall, in all secondary measures there was no difference between sertraline and placebo groups (**Table 3**), with the exception of MSEL fine motor age equivalent score, for which the mean improvement, adjusted for baseline score, was smaller for sertraline than placebo [mean difference: -2.9, 95% confidence interval (CI): -5.7 to -0.20]. Also, CGI-I at 3 months was examined as a *post hoc* analysis, and there was no difference between groups (F_1,48_ = 0.17, p = 0.866). Notably, for the aforementioned reasons for exclusion of data, usable PVET recordings were available for only 14 subjects (7 sertraline, 7 placebo) at both baseline and EOT. Repeated-measures ANOVA with treatment group as a between-subjects factor and time point as a within-subjects factor revealed no main effect of treatment group (F_1,12_ = 0.005, p = 0.94), no main effect of time point (F_1,12_ = 0.072, p = 0.79), and no interaction (F_1,12_ = 0.126, p = 0.73).

**Table 3:** Results of secondary measures.

Variables	Sertraline						Placebo						
	Baseline			End-of-treatment			Baseline			End-of-treatment			
	N	Mean	SD	N	Mean	SD	N	Mean	SD	N	Mean	SD	P-value, F
MSEL ELC standard score	32	56.66	11.41	26	56.31	12.28	26	58.88	14.44	21	60.95	19.72	0.0797, F_1,44_ = 3.22
PLS-5 total raw score	30	43.03	23.35	25	51.40	23.56	26	48.65	25.57	21	57.24	26.26	0.736, F_1,42_ = 0.12
CGI-I				26	1.23	1.11				21	1.14	0.96	0.7757, F_1,45_ = 0.08
VAS: A/OCB	32	4.87	2.58	26	6.48	2.61	25	4.98	2.30	20	6.86	2.37	0.7635, F_1,43_ = 0.09
VAS: L/C	31	2.03	1.70	25	4.70	2.73	25	2.84	2.04	21	5.53	2.95	0.9043, F_1,43_ = 0.01
VAS: A/H/H	32	4.52	2.51	26	5.64	2.71	26	4.30	2.49	21	6.61	2.46	0.2167, F_1,44_ = 1.57
VABS-II ABC	32	68.03	12.15	25	69.68	15.14	26	68.50	11.14	21	71.52	15.47	0.8691, F_1,43_ = 0.03
ABC-C composite score	32	57.25	29.58	26	49.85	32.69	25	57.96	24.15	21	43.91	24.99	0.2907, F_1,44_ = 1.14
PAS-R total raw score	32	14.75	11.58	26	14.77	14.45	24	15.38	12.51	21	11.43	10.04	0.1947, F_1,43_ = 1.74
SRS-2 total raw score	32	99.63	27.45	26	94.42	29.71	24	101.63	28.41	21	94.19	30.12	0.9072, F_1,43_ = 0.01
SPM-P total raw score	31	115.77	29.75	25	112.72	25.60	25	124.40	29.92	21	116.95	30.49	0.8745, F_1,43_ = 0.03

MSEL, Mullen Scales of Early Learning; ELC, Early Learning Composite; PLS-5, Preschool Language Scales, 5th edition; CGI-I, Clinical Global Impression Scale—Improvement; VAS, Visual Analog Scale (in units of centimeters); A/OCB, anxiety/obsessive compulsive behavior; L/C, language/communication; A/H/H, aggression/hyperarousal/hyperactivity; VABS-II ABC, Vineland-II Adaptive Behavior Composite; ABC-C: Aberrant Behavior Checklist—Community; PAS-R, Preschool Anxiety Scale—Revised; SRS-2, Social Responsiveness Scale; SPM-P, Sensory Processing Measure—Preschool.

Given the older average age of the sertraline group, age was incorporated as a covariate in the *post hoc* analysis of treatment effect. The results were consistent: treatment group difference was not significant for AEC score (F_1,43_ = 1.26, p = 0.267) and EL raw score (F_1,43_ = 0.28, p = 0.600). In both analyses, age was not associated with AEC score (F_1,43_ = 1.20, p = 0.280) and EL raw score (F_1,43_ = 3.04, p = 0.089).

### Correlation of Matrix Metalloproteinase-9 and Brain-Derived Neurotrophic Factor With Outcome

Higher baseline MMP9 was associated with lower MSEL AEC score (slope estimate = -0.95, F_1,39_ = 5.37, p = 0.026) in all subjects (sertraline and placebo); however, there was no difference in outcome between treatment groups across levels of MMP9 (F_1,39_ = 0.62, p = 0.437). Similarly, for MSEL EL raw score, there was no difference between groups at all levels of MMP9 (F_1,39_ = 0.06, p = 0.812), and MMP9 was not associated with outcome (F_1,39_ = 1.38, p = 0.247). Due to insufficient blood volume, MMP9 measurement at EOT was not available for analysis.

Baseline BDNF was not associated with MSEL AEC score (F_1,39_ = 3.55, p = 0.067) or EL raw score (F_1,39_ = 1.96, p = 0.170), and there was no difference in outcome between groups across all levels of baseline BDNF (F_1,39_ = 1.14, p = 0.293 and F_1,39_ = 2.40, p = 0.129 for MSEL AEC and EL raw scores, respectively). These results were the same for the secondary outcome of CGI-I at EOT, as well as for CGI-I at 3 months.

Further analyses were undertaken to examine whether changes in BDNF were related to improvement in outcomes. Change in BDNF was not associated with all outcomes, and there was no difference in outcomes between sertraline and placebo groups at all levels of change in BDNF. There was no difference in mean changes in BDNF levels between sertraline [mean 833.6 (SD 1081.2)] and placebo [mean 806.6 (SD 1578.7); t_36_ = 0.06, p = 0.952].

### Safety

There were 313 AEs reported. AEs were similar between treatment groups, with the top three most prevalent being upper respiratory infection (29 *vs*. 27%), diarrhea (7 *vs*. 12%), and hyperactivity (8 *vs*. 9%), for sertraline *vs*. placebo (**Table 4**). **Table 5** summarizes the characteristics of AEs by severity, relation to drug, status at study exit (resolved or ongoing), and serious AEs. One serious AE, hospitalization for dehydration secondary to viral infection, was reported in the sertraline group and resolved without sequelae or discontinuation of study treatment. No significant differences in characteristics of AEs were found between treatment groups: severity (Fisher’s exact test, p = 0.146) with mostly mild severity (88 *vs*. 87%, sertraline *vs*. placebo); relationship to drug (Fisher’s exact test, p = 0.641) with 55 *vs*. 58% not related to drug); and whether ongoing at study exit (any ongoing AE: 11 *vs*. 14%, Fisher’s exact test, p = 0.610).

**Table 4:** Types of adverse events (AEs).

	Sertraline		Placebo	
Adverse event (AE)	N	%	N	%
Upper respiratory infection (URI)	48	28.92	39	26.53
Diarrhea	11	6.63	17	11.56
Hyperactivity	14	8.43	13	8.84
Sleep disturbance	14	8.43	3	2.04
Irritability/aggression	9	5.42	11	7.48
Fever	10	6.02	8	5.44
Vomiting	9	5.42	3	2.04
Gastroenteritis	7	4.22	7	4.76
Ear infection	7	4.22	2	1.36
Rash	1	0.6	6	4.08
Lethargy	3	1.81	5	3.4
Tic	4	2.41	0	0
Constipation	2	1.2	3	2.04
Seasonal allergies	1	0.6	3	2.04
Decreased appetite	3	1.81	1	0.68
Dilated pupils	0	0	2	1.36
Eye redness	0	0	2	1.36
Obsessive behaviors	0	0	2	1.36
Self-injurious behavior	1	0.6	2	1.36
Encopresis	2	1.2	0	0
Increased stimming	2	1.2	1	0.68
Mouthing/chewing objects	2	1.2	0	0
Regression	2	1.2	0	0
Teeth grinding	2	1.2	1	0.68
Cut	0	0	1	0.68
Enuresis	1	0.6	1	0.68
Excessive drooling	0	0	1	0.68
Hand foot mouth disease	0	0	1	0.68
Headaches	0	0	1	0.68
Increase in pica	0	0	1	0.68
Itching	1	0.6	1	0.68
Localized left leg swelling after routine immunization	0	0	1	0.68
MRI finding: gray matter heterotopia	0	0	1	0.68
PE tube surgery	0	0	1	0.68
Possible seizure/syncope	0	0	1	0.68
Separation anxiety	1	0.6	1	0.68
Sprained ankle	0	0	1	0.68
Staph infection	0	0	1	0.68
Tuberous sclerosis	0	0	1	0.68
Urinary retention	0	0	1	0.68
Burned feet	1	0.6	0	0
Chicken pox	1	0.6	0	0
Flu	1	0.6	0	0
Gas	1	0.6	0	0
Hoarding	1	0.6	0	0
Inappropriate laughing	1	0.6	0	0
Inattention	1	0.6	0	0
Increased frequency of bowel movements	1	0.6	0	0
Tooth pain	1	0.6	0	0

MRI, magnetic resonance imaging.

**Table 5:** Characteristics of reported adverse events (AEs).

Variable	Category	Sertraline		Placebo		
		No. of AEs	%	No. of AEs	%	P-value^*^
**Severity**	Mild	146	87.95	128	87.07	0.1462
	Moderate	16	9.64	19	12.93	
	Severe	4	2.41	0	0	
**Relationship to drug**	Not related	91	54.82	85	57.82	0.6408
	Possibly related	64	38.55	56	38.1	
	Probably related	11	6.63	6	4.08	
**Serious AE**	No	165	99.4	147	100	1
	Yes	1	0.6	0	0	
**AE status at study exit**	Ongoing	19	11.45	20	13.61	0.6093
	Resolved	147	88.55	127	86.39	

*Test-statistic: Fisher’s exact test.

## Discussion

Previous studies suggest that some children with ASD may have low levels of serotonin during early development, due to either low serotonin production in the frontal cortex or deficits in the serotonin branch of the tryptophan metabolic pathway, or perhaps both (15, 16). Serotonin also appears to be implicated in the gastrointestinal dysfunction commonly seen in ASD: a mouse model possessing a serotonin transporter mutation (SERT Ala56), the most ubiquitous SERT mutation found in children with ASD, displays both behavioral and gastrointestinal manifestations of ASD, including a hypoplastic enteric nervous system with selective impairment in late-born neuronal development and survival (35). Treatment with the SSRI fluoxetine rescued this phenotype in mice (35). Case reports of children aged 6 to 12 years treated with low-dose fluoxetine cited rapid and significant improvement in self-injurious behavior, hyperactivity, irritability, and impulsivity (36). A recent national survey reported a caregiver-perceived overall net benefit from treatment with sertraline, though significant individual response variation was also reported (37). Taken together with evidence of greater benefit from targeted treatments during the sensitive developmental period, these data suggest the promise of SSRIs for treating symptoms of ASD in young children.

However, while several small studies in adults have shown promising results (26), Chugani et al. remains the only randomized controlled trial of a serotonin-influencing drug in young children with idiopathic ASD to date that suggested potential efficacy for this purpose (28). Prior to this 2016 trial of low-dose buspirone, five studies of treatment with SSRIs in children and adolescents with ASD, including a large randomized controlled trial of citalopram in individuals 5 to 17 years old, were included in a 2013 review concluding that there was insufficient evidence of SSRIs’ efficacy in children with ASD (26). However, none of these studies evaluated sertraline specifically. Thus, following the positive results of treatment with low-dose buspirone in ASD (28) and low-dose sertraline in FXS (29, 30), the authors undertook this study to ascertain whether sertraline was a beneficial treatment in non-FXS ASD.

**Figure 2:**
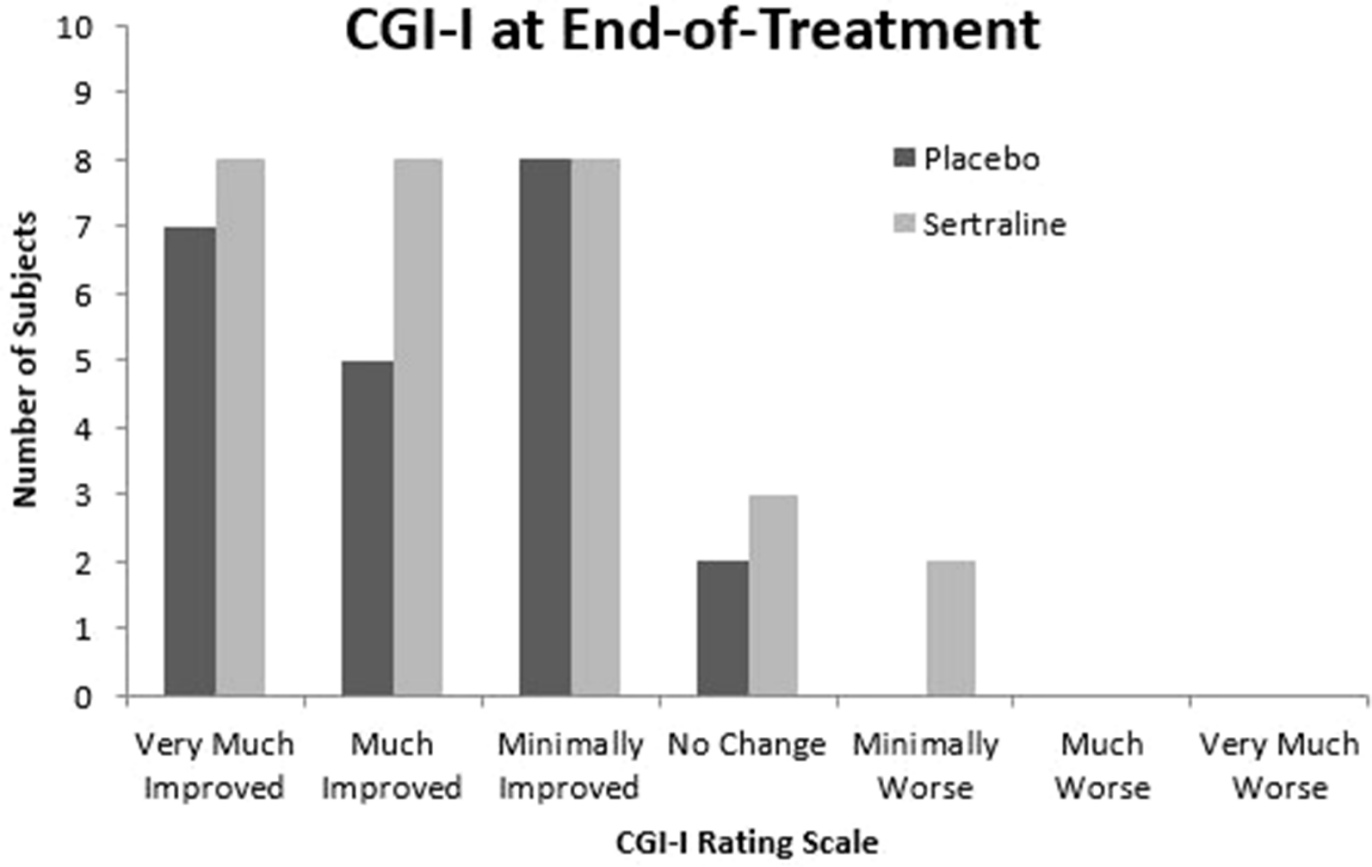
Clinical Global Impression Scale—Improvement at end-of-treatment. CGI-I, Clinical Global Impression Scale—Improvement.

The lack of efficacy seen in this study may be due, in part, to the extensively heterogenous behavioral manifestations, developmental trajectories, and myriad genetic factors at play in idiopathic or nonsyndromic ASD. Individual response to study treatment varied, and while certain subjects in the sertraline arm saw great improvement on the CGI-I, others did not benefit (**Figure 2**). There were no minimum or maximum verbal or other developmental requirements to qualify for inclusion in this study, which may have affected detectability of changes on the prespecified outcome measures. Additionally, although clinical genetic testing (other than *FMR1* testing) was not performed as part of this study, some caregivers voluntarily consented to add their children’s previous results of genetic or MRI testing to the study record. Of those subjects with available results, one had Dandy Walker Syndrome, one had Wiedemann Steiner Syndrome, one had a 15q13.3 microdeletion, one had 21q22.2 deletion, and one had MEF2C and PIK3C2G gene variants. All but one of these findings are known in the literature to be associated with higher rates of ASD than in the general population (38–41). Also, treatment response did not differentially follow either baseline BDNF or MMP9 levels, but other biomarkers could certainly be correlated. Therefore, genotypic or epigenotypic specificity in treatment response may explain the lack of efficacy observed in this trial.

This trial includes several limitations important to acknowledge, and the results should be considered preliminary. First, the relative size of this sample is small; there were significant challenges with subject recruitment and retention that may have affected the study’s reliability and power to detect efficacy. Many of the 179 caregivers contacted about the study declined to participate, expressing strong opposition to treating their young child with a psychotropic medication so early in life. The second most common reason for declining to participate was that the child was already being prescribed sertraline or another SSRI. The nature of the enrollment process thus selected for those caregivers who were both willing to give their child an off-label medication and comfortable with the possibility that their child would receive placebo. The authors speculate that this combination of factors may have biased the sample toward two extremes: children who were more severely affected, whose caregivers were more willing to consider experimental treatment, and children who were higher functioning, whose caregivers were more comfortable risking the chance of placebo.

Additionally, the trial drew subjects mainly from the northern California area, out of convenience. Thus, the study sample may not be representative of the greater population of children with ASD. Also, whenever possible, assessments were administered by the same rater at baseline and EOT, but in several instances, rater inconsistency nevertheless occurred and may have affected the results. The relatively high attrition rate observed in the study, coupled with the refusal of 11 out of 13 of these early terminated subjects to return for EOT assessments, is another limitation. Attrition and recruitment issues were likely seen in this study compared to the previous trial of sertraline in FXS due to the relative novelty of participating in clinical trials of investigational medications for the caregivers of children with ASD, whereas caregivers of those with FXS may be more readily accepting of treatment with psychotropic interventions early in childhood. Future trials of pharmacological interventions in young children with ASD could benefit from working with community stakeholders to maximize recruitment and retention.

Because the children in this study were followed for 6 months during a period in development normally characterized by language acquisition and skill development, the authors expected to observe significant developmental gains in both the sertraline and placebo arms. Thus, the improvement seen on the MSEL, the CGI-I, and other outcome measures in the placebo arm is largely explained by developmental changes that are typically anticipated at this age. Despite caregiver education regarding the placebo effect, there were several instances of caregiver ratings of improvement on the CGI-I that were not accompanied by significant increases in subjects’ scores on developmental assessments. Future studies in this population could benefit not only from selection of instruments that offer person ability scores that are designed for assessment of change, but also from a design that includes additional follow-up assessment time points to disentangle change from either measurement error or non-treatment-related developmental gains, though care should be taken to avoid possible practice effects.

In light of the lack of efficacy in this trial, the authors propose that a major factor in the positive response of children with both FXS and ASD in the previous controlled trial (29) was the heightened anxiety phenotype in the FXS subpopulation. Anxiety disorders, which normally affect 15–20% of youths without neurodevelopmental disorders, do occur at a high prevalence in individuals with ASD, with up to 37% of youth with ASD having been diagnosed specifically with social anxiety disorder, and even more (40-60%) meeting diagnostic criteria for an anxiety disorder of some kind (42–45). Among individuals with FXS, rates of social phobia reach between 35–60% and may be underestimated due to diagnostic criteria’s reliance on patient expression of “worry,” something not all individuals with FXS may do due to their intellectual disability (46). Prevalence of social anxiety in males with FXS and ASD is higher than that of those with FXS without ASD; specifically, initial social avoidance characterizes all males with FXS whereas prolonged social avoidance is associated only with the FXS with ASD phenotype (46, 47). Furthermore, while rates of anxiety may be comparably high between groups compared to typically developing controls, recent studies indicate that FXS and ASD may have distinct profiles for specific anxiety disorders; indeed, certain aspects of ASD symptomatology such as repetition of phrases or topics and gaze avoidance may actually reflect anxiety rather than social impairment in FXS, suggesting different underlying problems in FXS compared to nonsyndromic ASD (48, 49). The authors propose that these differences may result from a greater GABA deficit in FXS, leading to dampened ability to habituate to sensory stimuli and enhanced sympathetic responses to such stimuli, resulting in more intense anxiety (50). Regardless of the exact neurobiological mechanism, patients in the previous trial of sertraline with syndromic ASD were likely to have experienced greater relief from what appears to be a unique and heightened manifestation of anxiety compared to their peers with FXS or ASD alone, thereby facilitating behavioral or attentional improvements that led to greater developmental gains.

Importantly, the side effects of sertraline were not significantly different from placebo, and there was one serious AE in the sertraline arm that was not related to study treatment. Upon study completion, most caregivers opted to obtain a clinical prescription for sertraline for their child. Six-month treatment at this dose appears to be safe in young children with ASD, but follow-up would be essential, particularly if young patients continue on this medication for a longer duration.

## Conclusion

This preliminary controlled trial demonstrated no benefit of sertraline compared to placebo in young children with ASD. Future research directions include studies to replicate this trial’s findings, additional biomarker testing to examine treatment response variability, and long-term follow-up studies to assess the effects of low-dose sertraline use in young children later in development.

## Data Availability Statement

The datasets generated for this study are available on request to the corresponding author.

## Ethics Statement

The studies involving human participants were reviewed and approved by UC Davis Institutional Review Board (IRB) Administration, University of California, Davis Health System. Written informed consent to participate in this study was provided by the participants' legal guardian/next of kin.

## Author Contributions

RH, DS, FT, and SR contributed to the conception and design of the study; YC and PD performed the statistical analyses; DN contributed to study design, analysis and writing of the manuscript. LP wrote the first draft of the manuscript; AS administered developmental and language assessments; and NC and RO processed and analyzed biomarkers data. All authors contributed to manuscript writing and revision, and all authors read and approved the submitted version.

## Funding

This project was supported by the Health Resources and Services Administration (HRSA) of the U.S. Department of Health and Human Services (HHS) under grant number R40MCH 27701. This information or content and conclusions are those of the author and should not be construed as the official position or policy of, nor should any endorsements be inferred by HRSA, HHS or the U.S. Government. Other support includes the MIND Institute Intellectual and Developmental Disabilities Research Center which is funded by the National Institute of Child Health and Human Development (U54 HD079125). This publication was also made possible by National Center for Advancing Translational Sciences and National Institutes of Health (grant UL1 TR001860).

## Conflict of Interest

RH has carried out treatment studies in fragile X syndrome and autism spectrum disorder by Roche, Novartis, Neuren, Marinus, Alcobra, and Curemark and has also consulted with Zynerba and Fulcrum. FT received funds from Asuragen, Roche, and Zynerba.

The remaining authors declare that the research was conducted in the absence of any commercial or financial relationships that could be construed as a potential conflict of interest.

## References

[1] Youssef EA Berry-Kravis E Czech C Hagerman RJ Hessl D Wong CY Effect of the mGluR5-NAM Basimglurant on Behavior in Adolescents and Adults with Fragile X Syndrome in a Randomized, Double-Blind, Placebo-Controlled Trial: FragXis Phase 2 Results. Neuropsychopharmacology (2018) 43(3):503–12. 10.1038/npp.2017.177 PMC5770759 28816242

[2] De Rubeis S He X Goldberg AP Poultney CS Samocha K Cicek AE Synaptic, transcriptional and chromatin genes disrupted in autism. Nature (2014) 515(7526):209–15. 10.1038/nature13772 PMC4402723 25363760

[3] Baio J Wiggins L Christensen DL Maenner MJ Daniels J Warren Z Prevalence of Autism Spectrum Disorder Among Children Aged 8 Years - Autism and Developmental Disabilities Monitoring Network, 11 Sites, United States, 2014. Mmwr Surveill Summ (2018) 67(6):1–23. 10.15585/mmwr.ss6706a1 PMC5919599 29701730

[4] Iossifov I O'Roak BJ Sanders SJ Ronemus M Krumm N Levy D The contribution of de novo coding mutations to autism spectrum disorder. Nature (2014) 515:216. 10.1038/nature13908 25363768 PMC4313871

[5] Iossifov I Ronemus M Levy D Wang Z Hakker I Rosenbaum J De novo gene disruptions in children on the autistic spectrum. Neuron (2012) 74(2):285–99. 10.1016/j.neuron.2012.04.009 PMC3619976 22542183

[6] Lo SQ Sng JCG Augustine GJ Defining a critical period for inhibitory circuits within the somatosensory cortex. Sci Rep (2017) 7(1):7271–. 10.1038/s41598-017-07400-8 28779074 PMC5544762

[7] Reinhard SM Razak K Ethell IM A delicate balance: role of MMP-9 in brain development and pathophysiology of neurodevelopmental disorders. Front Cell Neurosci (2015) 9:280–. 10.3389/fncel.2015.00280 26283917 PMC4518323

[8] Su T Fan H-X Jiang T Sun W-W Den W-Y Gao M-M Early continuous inhibition of group 1 mGlu signaling partially rescues dendritic spine abnormalities in the Fmr1 knockout mouse model for fragile X syndrome. Psychopharmacology (2011) 215(2):291–300. 10.1007/s00213-010-2130-2 21181121

[9] Ismail FY Fatemi A Johnston MV Cerebral plasticity: Windows of opportunity in the developing brain. Eur J Paediatric Neurology (2017) 21(1):23–48. 10.1016/j.ejpn.2016.07.007 27567276

[10] Meredith RM Sensitive and critical periods during neurotypical and aberrant neurodevelopment: A framework for neurodevelopmental disorders. Neurosci Biobehavioral Rev (2015) 50:180–8. 10.1016/j.neubiorev.2014.12.001 25496903

[11] LeBlanc JJ Fagiolini M Autism: a "critical period" disorder? Neural plasticity (2011) 2011:921680–. 10.1155/2011/921680 21826280 PMC3150222

[12] He Q Arroyo ED Smukowski SN Xu J Piochon C Savas JN Mol Psychiatry (2018). 10.1038/s41380-018-0048-y PMC6204122 29703945

[13] Dawson G Rogers S Munson J Smith M Winter J Greenson J Randomized, controlled trial of an intervention for toddlers with autism: the Early Start Denver Model. Pediatrics (2010) 125(1):e17–23. 10.1542/peds.2009-0958 PMC4951085 19948568

[14] Dawson G Jones EJH Merkle K Venema K Lowy R Faja S Early behavioral intervention is associated with normalized brain activity in young children with autism. J Am Acad Child Adolesc Psychiatry (2012) 51(11):1150–9. 10.1016/j.jaac.2012.08.018 PMC3607427 23101741

[15] Chandana SR Behen ME Juhasz C Muzik O Rothermel RD Mangner TJ Significance of abnormalities in developmental trajectory and asymmetry of cortical serotonin synthesis in autism. Int J Dev Neurosci (2005) 23(2-3):171–82. 10.1016/j.ijdevneu.2004.08.002 15749243

[16] Boccuto L Chen CF Pittman AR Skinner CD McCartney HJ Jones K Decreased tryptophan metabolism in patients with autism spectrum disorders. Mol Autism (2013) 4(1):16. 10.1186/2040-2392-4-16 23731516 PMC3680090

[17] Bianchi P Ciani E Guidi S Trazzi S Felice D Grossi G Early pharmacotherapy restores neurogenesis and cognitive performance in the Ts65Dn mouse model for Down syndrome. J Neurosci (2010) 30(26):8769–79. 10.1523/JNEUROSCI.0534-10.2010 PMC6632890 20592198

[18] Mei F Nagappan G Ke Y Sacktor TC Lu B BDNF Facilitates L-LTP Maintenance in the Absence of Protein Synthesis through PKMζ. PloS One (2011) 6(6):e21568. 10.1371/journal.pone.0021568 21747912 PMC3126837

[19] Greenberg ME Xu B Lu B Hempstead BL New insights in the biology of BDNF synthesis and release: implications in CNS function. J Neurosci: Off J Soc Neurosci (2009) 29(41):12764–7. 10.1523/JNEUROSCI.3566-09.2009 PMC3091387 19828787

[20] Qin XY Cheng Y Brain-Derived Neurotrophic Factor in Autism Spectrum Disorder-Reply. JAMA Pediatr (2017) 171(5):493. 10.1001/jamapediatrics.2017.0110 28264085

[21] Jansson LC Louhivuori L Wigren HK Nordström T Louhivuori V Castrén ML Brain-derived neurotrophic factor increases the motility of a particular N-methyl-d-aspartate /GABA-responsive subset of neural progenitor cells. Neuroscience (2012) 224:223–34. 10.1016/j.neuroscience.2012.08.038 22922352

[22] Lauterborn JC Rex CS Kramár E Chen LY Pandyarajan V Lynch G Brain-Derived Neurotrophic Factor Rescues Synaptic Plasticity in a Mouse Model of Fragile X Syndrome. J Neurosci (2007) 27(40):10685–94. 10.1523/JNEUROSCI.2624-07.2007 PMC6672822 17913902

[23] Abdallah MW Michel TM Matrix metalloproteinases in autism spectrum disorders. J Mol Psychiatry (2013) 1(1):16. 10.1186/2049-9256-1-16 25408909 PMC4223892

[24] Hasan M Seo JE Rahaman KA Kang MJ Jung BH Kwon OS Increased levels of brain serotonin correlated with MMP-9 activity and IL-4 levels resulted in severe experimental autoimmune encephalomyelitis (EAE) in obese mice. Neuroscience (2016) 319:168–82. 10.1016/j.neuroscience.2016.01.045 26820599

[25] Saghazadeh A Rezaei N Brain-Derived neurotrophic factor levels in autism: a systematic review and meta-analysis. J Autism Dev Disord (2017) 47(4):1018–29. 10.1007/s10803-016-3024-x 28138831

[26] Williams K Brignell A Randall M Silove N Hazell P Selective serotonin reuptake inhibitors (SSRIs) for autism spectrum disorders (ASD). Cochrane Database systematic Rev (2013) (8), Cd004677. 10.1002/14651858.CD004677.pub3. PMC11990048 23959778

[27] Winarni TI Schneider A Borodyanskara M Hagerman RJ Early intervention combined with targeted treatment promotes cognitive and behavioral improvements in young children with fragile x syndrome. Case Rep Genet (2012) 2012:280813. 10.1155/2012/280813 23074686 PMC3447258

[28] Chugani DC Chugani HT Wiznitzer M Parikh S Evans PA Hansen RL Efficacy of Low-Dose Buspirone for Restricted and Repetitive Behavior in Young Children with Autism Spectrum Disorder: A Randomized Trial. J Pediatr (2016) 170:45–53. 10.1016/j.jpeds.2015.11.033 26746121

[29] Greiss Hess L Fitzpatrick SE Nguyen DV Chen Y Gaul KN Schneider A A Randomized, Double-Blind, Placebo-Controlled Trial of Low-Dose Sertraline in Young Children With Fragile X Syndrome. J Dev Behav Pediatr (2016) 37(8):619–28. 10.1097/DBP.0000000000000334 PMC5039060 27560971

[30] Yoo K Burris J Gaul K Hagerman R Rivera S Low-Dose Sertraline Improves Receptive Language in Children with Fragile X Syndrome when Eye Tracking Methodology is used to Measure Treatment Outcome. J Psychol Clin Psychiatry (2017) 7(6). 10.15406/jpcpy.2017.07.00465.

[31] AlOlaby RR Sweha SR Silva M Durbin-Johnson B Yrigollen CM Pretto D Molecular biomarkers predictive of sertraline treatment response in young children with fragile X syndrome. Brain Dev (2017) 39(6):483–92. 10.1016/j.braindev.2017.01.012 PMC5420478 28242040

[32] Tassone F Choudhary NS Tassone F Durbin-Johnson B Hansen R Hertz-Picciotto I Identification of expanded alleles of the FMR1 Gene in the CHildhood Autism Risks from Genes and Environment (CHARGE) study. J Autism Dev Disord (2013) 43(3):530–9. 10.1007/s10803-012-1580-2 PMC4596818 22767137

[33] Dink J Ferguson B, (2015). eyetrackingR: An R Library for Eye-Tracking Data Analysis.

[34] Hoenig JM Heisey DM The Abuse of Power. Am Statistician (2001) 55(1):19–24. 10.1198/000313001300339897

[35] Margolis KG Li Z Stevanovic K Saurman V Israelyan N Anderson GM Serotonin transporter variant drives preventable gastrointestinal abnormalities in development and function. J Clin Invest (2016) 126(6):2221–35. 10.1172/JCI84877 PMC4887174 27111230

[36] Lucchelli JP Bertschy G Low-Dose Fluoxetine in Four Children with Autistic Spectrum Disorder Improves Self-Injurious Behavior, ADHD-Like Symptoms, and Irritability. Case Rep Psychiatry (2018) 2018:6278501–. 10.1155/2018/6278501 30002940 PMC5998188

[37] Coleman DM Adams JB Anderson AL Frye RE Rating of the Effectiveness of 26 Psychiatric and Seizure Medications for Autism Spectrum Disorder: Results of a National Survey. J Child Adolesc Psychopharmacology (2019) 29(2):107–23. 10.1089/cap.2018.0121 PMC6442266 30724573

[38] Basson MA Wingate RJ Congenital hypoplasia of the cerebellum: developmental causes and behavioral consequences. Front neuroanatomy (2013) 7:29–. 10.3389/fnana.2013.00029 PMC3759752 24027500

[39] Baer S Afenjar A Smol T Piton A Gérard B Alembik Y Wiedemann-Steiner syndrome as a major cause of syndromic intellectual disability: a study of 33 French cases. Clin Genet (2018) 94(1):141–52. 10.1111/cge.13254 29574747

[40] Ziats MN Goin-Kochel RP Berry LN Ali M Ge J Guffey D The complex behavioral phenotype of 15q13.3 microdeletion syndrome. Genet In Med (2016) 18:1111. 10.1038/gim.2016.9 26963284

[41] Wenger TL Miller JS DePolo LM de Marchena AB Clements CC Emanuel BS 22q11.2 duplication syndrome: elevated rate of autism spectrum disorder and need for medical screening. Mol Autism (2016) 7:27. 10.1186/s13229-016-0090-z 27158440 PMC4859984

[42] Beesdo K Knappe S Pine DS Anxiety and anxiety disorders in children and adolescents: developmental issues and implications for DSM-V. Psychiatr Clinics North America (2009) 32(3):483–524. 10.1016/j.psc.2009.06.002 PMC3018839 19716988

[43] Salum GA Desousa DA Rosario MC Pine DS Manfro GG Pediatric anxiety disorders: from neuroscience to evidence-based clinical practice. In: Revista brasileira de psiquiatria., vol. 35 Suppl 1 , Sao Paulo, Brazil (2013). p. S03–21. 10.1590/1516-4446-2013-S108. 24142122

[44] White SW Oswald D Ollendick T Scahill L Anxiety in children and adolescents with autism spectrum disorders. Clin Psychol review (2009) 29(3):216–29. 10.1016/j.cpr.2009.01.003 PMC2692135 19223098

[45] Kerns CM Kendall PC Berry L Souders MC Franklin ME Schultz RT Traditional and atypical presentations of anxiety in youth with autism spectrum disorder. J Autism Dev Disord (2014) 44(11):2851–61. 10.1007/s10803-014-2141-7 PMC5441227 24902932

[46] Cordeiro L Ballinger E Hagerman R Hessl D Clinical assessment of DSM-IV anxiety disorders in fragile X syndrome: prevalence and characterization. J Neurodev Disord (2011) 3(1):57–67. 10.1007/s11689-010-9067-y 21475730 PMC3057014

[47] Roberts JE Ezell JE Fairchild AJ Klusek J Thurman AJ McDuffie A Biobehavioral composite of social aspects of anxiety in young adults with fragile X syndrome contrasted to autism spectrum disorder. Am J Med Genet Part B: Neuropsychiatr Genet (2018) 177(7):665–75. 10.1002/ajmg.b.32674 PMC6532983 30307687

[48] Ezell J Hogan A Fairchild A Hills K Klusek J Abbeduto L Prevalence and Predictors of Anxiety Disorders in Adolescent and Adult Males with Autism Spectrum Disorder and Fragile X Syndrome. J Autism Dev Disord (2018) 49(3):1131–41. 10.1007/s10803-018-3804-6 PMC6596989 30430320

[49] Abbeduto L Thurman AJ McDuffie A Klusek J Feigles RT Ted Brown W ASD Comorbidity in Fragile X Syndrome: Symptom Profile and Predictors of Symptom Severity in Adolescent and Young Adult Males. J Autism Dev Disord (2019) 49(3):960–77. 10.1007/s10803-018-3796-2 PMC6556533 30382442

[50] Miller LJ McIntosh DN McGrath J Shyu V Lampe M Taylor AK Electrodermal responses to sensory stimuli in individuals with fragile X syndrome: a preliminary report. Am J Med Genet (1999) 83(4):268–79. 10.1002/(SICI)1096-8628(19990402)83:4<268::AID-AJMG7>3.0.CO;2-K 10208160

